# The Needs of Wounded Women

**Published:** 1914-10-17

**Authors:** K. Harvey


*To the Editor of* THE HOSPITAL.

### The Needs of Wounded Women.

*To the Editor of* THE HOSPITAL.

SIR,—We honour and are proud beyond words of our wounded men, but that must not make us overlook our wounded women, wounded in the battle of life, and *for* life; we must not forget that responsibility, overwork, insufficient food, and anxiety will increase their usual average of sickness, which is always large during the wet and cold season.

The heavy death of young and healthy men must seriously affect the coming generation, therefore it becomes a national duty, of more than ordinary importance, to keep the mothers—actual and potential—in a high state of efficiency, physically and mentally, and the children robust, for in them, more than ever, lies our hope for the life of the world to come. Not one woman or child should be denied medical aid and nursing if within human power to give them the help they need; to handicap them in the matter of health, if by any means we can prevent it, would be national suicide. Civilian patients are already suffering severely from shortage of hospital beds, doctors, and nurses. At one hospital alone there are one hundred women waiting for admission. Urgent cases have been refused help, and children cannot be sent to convalescent homes, because they are being reserved for the soldiers. My knowledge has all been obtained first hand, and I am ready to give full information to anyone who likes to call upon me. It is playing with words to argue that, in this way, the war is entailing no extra suffering, because it is the logical outcome of the situation. Taking hospitals all round, there cannot be as many beds as usual at the disposal of civilians; this must raise the "waiting" average somewhere, and the trouble will get rapidly worse.

If common sense bids us, for national preservation, send our men to the Front, it also bids us keep our women strong, so that their superabundant health may counteract as far as possible one evil effect of war.

I intend to run my house at Bromley, Kent, on these lines, and will make all necessary preparations to that end as a "sample" of what can be done, hoping that the scheme will take hold of the public conscience. But I cannot run a hospital personally long enough to make the expense worth while unless I can obtain financial support.

I propose taking: Maternity cases, after their dismissal at the end of ten days; surgical operation cases which have to leave the hospital before any real approach to health has been attained; children who cannot be admitted to the convalescent homes.

This work is undoubtedly "National Service," also "relief for soldiers and sailors," for I am much mistaken in "Tommy Atkins" and "Jack" if it will not afford them the greatest relief to know that their women and children are being well and systematically cared for.

There is no reason why my scheme should not be carried out in other towns, and so relieve the congestion at the London hospitals and enable them to deal with many more acute cases. Local doctors could give their services so as to encroach upon their time as little as possible; local nurses from hospitals, nursing homes, etc., could take short shifts so that no one could be overtaxed; chemists could supply medicine, etc., at cost price; and local tradesmen could help, at any rate in kind.

All mechanical work, such as washing patients, taking temperature, etc., could be done by those women who have offered their services for wounded men: it would keep them in practice for actual war work.

For the future welfare of the nation I beg you will kindly give this scheme as much publicity as possible.

It would greatly increase the efficiency of the work to have a permanent fully qualified nurse. Will those sympathisers who cannot give any large sum contribute weekly one shilling towards a "nurse's fee fund"?

Donations may be paid direct to Lloyd's Bank, Limited, Bromley, for the credit of "Brackenhill Hospital" account, or sent to me personally—and every subscriber of £5 or over will receive a monthly memorandum of expenditure.—I am, etc., (Mrs.) K. HARVEY.

